# Supplementary material for: On the micelle formation of DNAJB6b
Source: QRB Discov. 2023 Aug 15;4:e6. doi: 10.1017/qrd.2023.4 (PMC10427797; doi:10.1017/qrd.2023.4)
Supplement: Supplementary file 1 [file S2633289223000042sup001.pdf]

Supplementary Information to "On the Micelle Formation of DNAJB6b"

Andreas Carlsson[1*], Ulf Olsson[2] and Sara Linse[1]

[1]Biochemistry and Structural Biology, Chemical Centre, Lund University, Lund, Sweden
[2]Physical Chemistry, Chemical Centre, Lund University, Lund, Sweden
*Andreas Carlsson, E-mail: andreas.carlsson@biochemistry.lu.se

Different flowrates were used when using MDS, which was needed to cover a broad range of detectable sizes. Furthermore, the Alexa647-labelled series were measured after five and nine days, to validate that the equilibrium times obtained from Figure 3 are valid at various protein concentrations. The mean of all flowrates and measuring days, for each protein concentration, are showed in Figure 4. The data used to calculate the mean values are showed in Figure S1.

There is no clear size difference between five and nine days. There might be a slight variation between various flowrates, which is to be expected when analyzing samples containing highly different sizes, such as various oligomeric states of DNAJB6b.

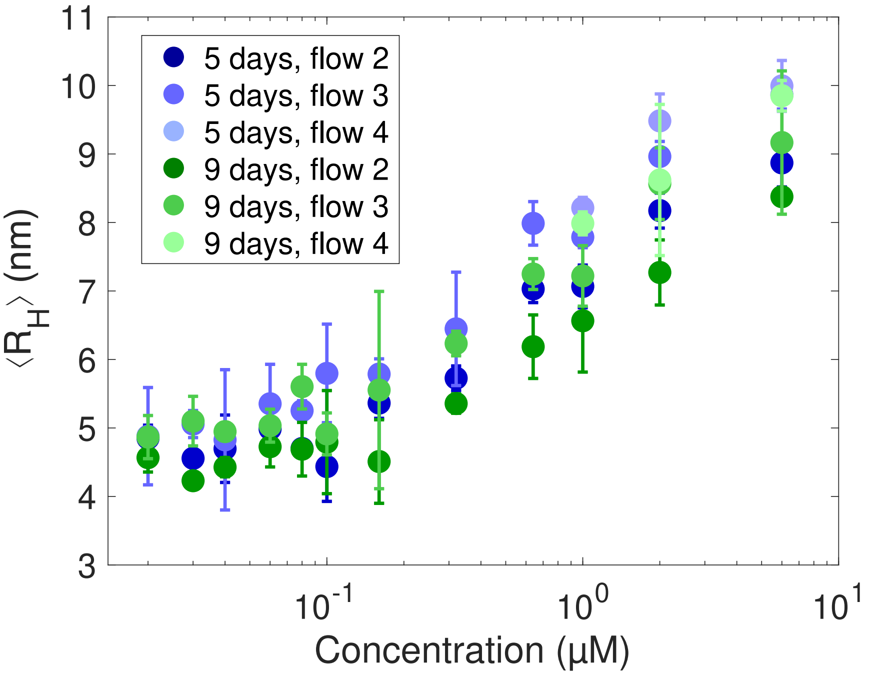

Figure 1: The data used to obtain the data points of the lower concentrations in Figure 4, Alexa647-labelled series. Triplicate of each sample, flowrate and day. The errorbars represent standard deviations.
